# Malignant adenomyoepithelioma of the breast: A case report

**DOI:** 10.1097/MD.0000000000024461

**Published:** 2021-02-05

**Authors:** Zhihao Zhang, Yueyuan Wang, Xiao Xie, Jingyu Peng, Jinghui Hong, Lirong Bi, Ming Yang

**Affiliations:** a Department of Breast Surgery; b Department of Pathology, First Hospital of Jilin University, Changchun, People's Republic of China.

**Keywords:** adenomyoepithelioma, breast, malignant, myoepithelium

## Abstract

**Rationale:**

Adenomyoepithelioma (AME) is a rare biphasic tumor consisting of epithelial and Myoepithelial cell. Most of the AME is benign, and only a few will progress to malignancy, Here, we report a case of low-grade malignant adenomyoepithelioma, and review the related literature, in a bid to investigate its clinical and pathological features and thus, enhance our understanding of this tumor.

**Patient concerns:**

A 64-year-old woman visited our hospital with a 1-year history of a painless mass in her left breast. Physical examination revealed a palpable painless mass, measuring approximately 4.5 cm, in the left breast.

**Diagnosis:**

Histological examination confirmed the diagnosis of malignant adenomyoepithelioma

**Interventions:**

The patient underwent local excision of the mass, with frozen section analysis revealing ductal carcinoma in situ. Mastectomy and sentinel lymph node biopsy were then performed.

**Outcomes:**

We conducted a one-year follow-up, and relapse was not observed.

**Lessons:**

Treatment of AME remains controversial owing to the lack of high volume data and absence of prospective studies. Simple mastectomy is an acceptable treatment of this tumor.

## 1 Introduction

Adenomyoepithelioma (AME) was first reported by Hamperl^[1]^ in 1970. In 2012, the World Health Organization (WHO) classified malignant adenomyoepithelioma (MAME) as an “adenomyoepitheliomatous variant,” including carcinomas of epithelial, myogenic, and epithelial-myogenic origin.^[2]^ Because of the heterogeneity of AME, the diagnosis is challenging and a range of histological patterns can be observed. These lesions are particularly common in patients undergoing core needle biopsy. While most of AME is benign, there have been reports of distant metastasis. All the reported malignant AMEs with metastases have shown significant cytological atypia and brisk mitotic rates. T Therefore, a complete resection of the tissue is necessary for pathological diagnosis.

## 2 Case report

**Figure 1:**
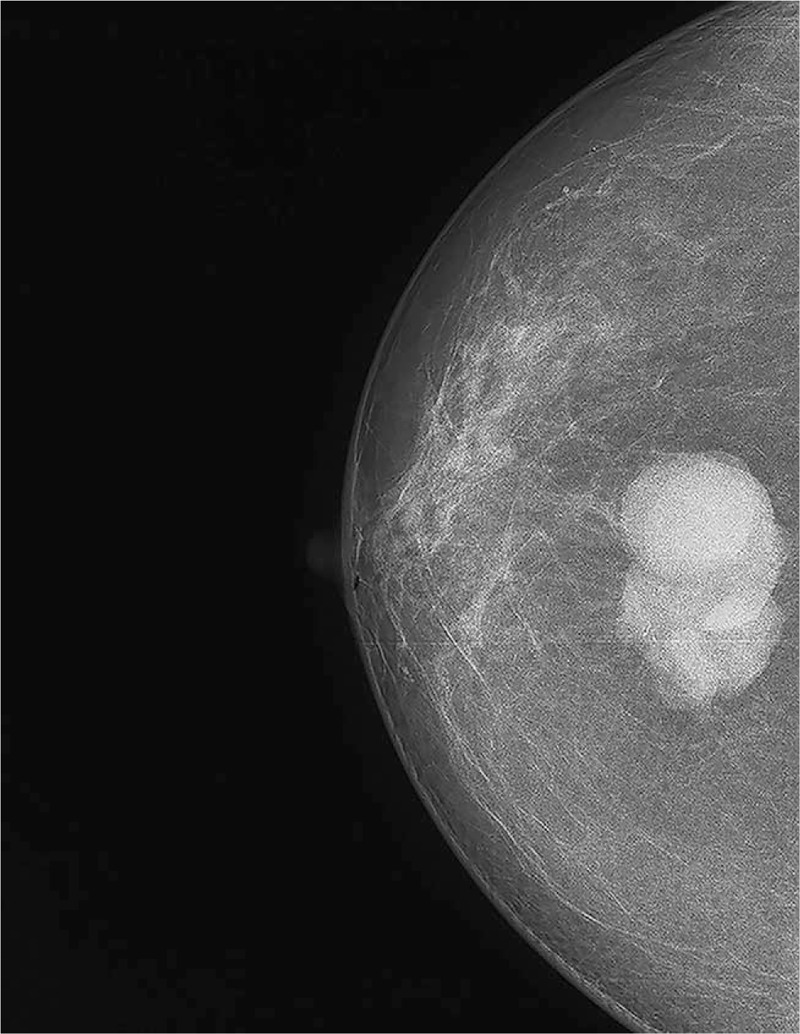
Mammography scan showing a 40 × 40 mm nodule with unclear margins and no calcification observed within the mass.

**Figure 2:**
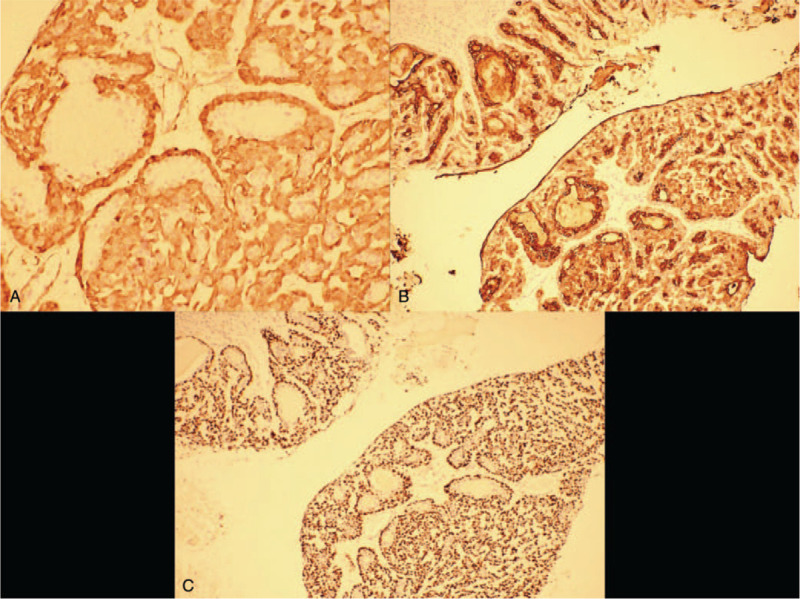
A, Immunohistochemical staining of the resected tissue. Positive calponin staining in myoepithelial cells (200 × magnification). B, Immunohistochemical staining of the resected tissue. Myoepithelial cells were very strongly positive for cytokeratin-5 (CK5) (100 × magnification). C, Immunohistochemical staining of the resected tissue. Nuclear P63 staining in the myoepithelial component confirmed differentiation of the myoepithelium (100 × magnification).

A 64-year-old woman visited our hospital with a 1-year history of a painless mass in her left breast. The patient reported rapid mass growth over the previous 6 months. Physical examination detected a palpable painless mass, measuring approximately 4.5 cm, in the left breast. Ultrasonography revealed a hypoechoic lump measuring 39.4 mm × 22.6 mm at 6 o’clock in the left breast with poorly defined borders. Mammography revealed a nodule of approximately 40 mm × 40 mm in size with poorly defined borders and no calcification visible in the left breast (Fig. 1). The patient underwent local excision of the mass, and frozen section analysis revealed ductal carcinoma in situ. Subsequently, mastectomy and sentinel lymph node biopsy were performed. Definitive histological examination diagnosed low-grade MAME, with local microinvasion (0.1 cm), malignant degeneration of the myoepithelium in the tumor, and easily visible mitotic figures (local foci >10/10 HPF). Further, intraoperative examination (left sentinel lymph node) did not detect any cancer metastasis by serial sections (0/3). Immunohistochemical results were as follows: ER (−), PR (−), Her-2 (0), S-100 (+), Calponin (+), CK5/6 (+), and P63 (+) (Fig. 2A–C). No further treatment was administered. We conducted a 1-year follow-up, and the patient showed no signs of relapse.

## 3 Discussion

AME is a rare tumor composed of myoepithelial and epithelial cells, whereas MAME is even more uncommon. There are less than 20 reports on MAME and most literature reports on female cases.^[3]^ The onset age of MAME is between 26 and 81 years, and the incidence increases with age.^[4]^ Painless breast lumps are common in clinical practice, with no lymph node involvement. In mammography, tumors tend to appear well defined, dense, and calcified. Ultrasonography often reveals well-defined, homogeneous solid nodules or cystic solid lesions.^[5,6]^

Although AMEs are commonly benign, they must be considered malignant when they present with cytological atypia, an infiltrative growth pattern, and a high mitotic index (local >3/10 HPF).^[7]^ The cellular composition of MAMEs was confirmed by immunolabeling, with epithelial elements typically showing positive staining for low molecular weight cytokeratin, including CAM 5.2, CK7, and EMA. In contrast, positive staining for high molecular weight cytokeratin, such as CK5/6, was characteristic of myoepithelial components. In addition, myoepithelial markers also included S100, SMA, and p63.^[3,5,8,9]^

Treatment of AME remains controversial owing to the lack of high volume data and absence of prospective studies. Based on the available evidence, benign AMEs may only require complete surgical resection, whereas malignant tumors involving the breast and/or regional lymphatics could respond to radiotherapy.^[7,9,10]^ Whether chemotherapy is effective for MAME is unclear owing to the paucity of literature. However, rare instances of metastatic disease would presumably require chemotherapy, and studies of targeted anti-cancer therapy have also been presented in case reports.^[11]^ Routine use of sentinel lymph node biopsy may not be necessary compared to invasive breast cancer.^[12]^ Therefore, there is currently no standardized treatment for MAME.

It is challenging for us to assess the prognosis of MAME owing to the low volume of data. Nevertheless, a retrospective study of 110 patients evaluated the prognosis of MAME. The study indicated that the expected 5-year OS for all patients of MAME was 74.4%.^[12]^

## 4 Conclusion

In conclusion, treatment of AME remains controversial owing to the lack of high volume data and absence of prospective studies. Simple mastectomy is increasingly accepted by clinicians. In addition, MAME should be distinguished from other breast diseases, such as myoepithelial carcinoma, mammary gland sarcoma, invasive breast cancer, tubular adenoma, and intraductal papilloma, among others.^[4,9]^ MAME has the possibility of distant metastasis and recurrence. Therefore, it is imperative for clinical pathologists to monitor the progress of MAME.

## Author contributions

**Conceptualization:** Xiao Xie.

**Data curation:** Zhihao Zhang, Jingyu Peng.

**Investigation:** Xue Wei, Yueyuan Wang, Jinghui Hong.

**Supervision:** Ming Yang.

**Visualization:** Lirong Bi.

**Writing – original draft:** Zhihao Zhang.

**Writing – review & editing:** Ming Yang.

## References

[1] Hamperl h The myothelia (myoepithelial cells). Normal state; regressive changes; hyperplasia; tumors. Curr Top Pathol 1970;53:161–220. 4323195

[2] Tan PH Ellis IO Myoepithelial and epithelial-myoepithelial, mesenchymal and fibroepithelial breast lesions: updates from the WHO Classification of Tumours of the Breast 2012. J Clin Pathol 2013;66:465–70. 23533258 10.1136/jclinpath-2012-201078

[3] Jones M Fletcher J Malignant adenomyoepithelioma of the breast. Pathology 2017;49:322–5. 28249686 10.1016/j.pathol.2016.11.015

[4] Intagliata e Gangi s Trovato c Benign adenomyoepitelioma of the breast: Presentation of two rare cases and review of literature. Int J Surg Case Rep 2020;67:1–4. 31991375 10.1016/j.ijscr.2020.01.010 PMC6992528

[5] Gafton B Scripcariu V Prutianu I Challenges in management of male breast adenomioepithelioma with malignant behavior: case report. Medicine 2019;98:e17587. 31651866 10.1097/MD.0000000000017587 PMC6824645

[6] Yuan Z Qu X Zhang ZT Lessons from managing the breast malignant adenomyoepithelioma and the discussion on treatment strategy. World J Oncol 2017;8:126–31. 29147448 10.14740/wjon1055e PMC5650010

[7] Wiens n Hoffman dI Huang cY Clinical characteristics and outcomes of benign, atypical, and malignant breast adenomyoepithelioma: a single institution's experience. Am J Surg 2020;219:651–4. 30982573 10.1016/j.amjsurg.2019.03.026

[8] Yoon jY Chitale d Adenomyoepithelioma of the breast: a brief diagnostic review. Arch Pathol Lab Med 2013;137:725–9. 23627458 10.5858/arpa.2011-0404-RS

[9] Logie N Hugh J Paulson K Radiotherapy in the multidisciplinary management of adenomyoepithelioma of the breast with an axillary lymph node metastasis: a case report and review of the literature. Cureus 2017;9:e1380. 28775920 10.7759/cureus.1380 PMC5522019

[10] Lee s Oh sY Kim sH Malignant Adenomyoepithelioma of the Breast and Responsiveness to Eribulin. J Breast Cancer 2015;18:400–3. 26770248 10.4048/jbc.2015.18.4.400 PMC4705093

[11] Hikino H Nagaoka S Miura H Benign myoepithelioma of the breast: origin and development. Pathol Int 2009;59:422–6. 19490475 10.1111/j.1440-1827.2009.02388.x

[12] Haque W Verma V Suzanne Klimberg V Clinical presentation, national practice patterns, and outcomes of breast adenomyoepithelioma. Breast J 2020;26:653–60. 31578797 10.1111/tbj.13638

